# Generation of Breast Cancer Stem Cells by Steroid Hormones in Irradiated Human Mammary Cell Lines

**DOI:** 10.1371/journal.pone.0077124

**Published:** 2013-10-16

**Authors:** Guillaume Vares, Xing Cui, Bing Wang, Tetsuo Nakajima, Mitsuru Nenoi

**Affiliations:** 1 Research Center for Radiation Protection, National Institute of Radiological Sciences, Chiba, Japan; 2 Research Center for Charged Particle Therapy, National Institute of Radiological Sciences, Chiba, Japan; Northwestern University Feinberg School of Medicine, United States of America

## Abstract

Exposure to ionizing radiation was shown to result in an increased risk of breast cancer. There is strong evidence that steroid hormones influence radiosensitivity and breast cancer risk. Tumors may be initiated by a small subpopulation of cancer stem cells (CSCs). In order to assess whether the modulation of radiation-induced breast cancer risk by steroid hormones could involve CSCs, we measured by flow cytometry the proportion of CSCs in irradiated breast cancer cell lines after progesterone and estrogen treatment. Progesterone stimulated the expansion of the CSC compartment both in progesterone receptor (PR)-positive breast cancer cells and in PR-negative normal cells. In MCF10A normal epithelial PR-negative cells, progesterone-treatment and irradiation triggered cancer and stemness-associated microRNA regulations (such as the downregulation of miR-22 and miR-29c expression), which resulted in increased proportions of radiation-resistant tumor-initiating CSCs.

## Introduction

Worldwide, breast cancer represents 16% of all cancer incidence among women and 13.7% of cancer deaths [1]. It was shown that women who had received medium or high-dose ionizing radiation to the chest (for example, as treatments for other cancers, such as Hodgkin’s lymphoma) have a relative risk of breast cancer between 2.1 and 4.0 [2]. By age 45 years, up to 20% of women exposed to chest ionizing radiation for a pediatric malignancy are diagnosed with breast cancer [3]. New data are coming to light indicating that even low dose exposures (such as diagnostic chest X-rays for tuberculosis or pneumonia) might raise this risk [4]. Sex steroid hormones such as estrogen and progesterone play a crucial role in the development and homeostasis of the mammary gland, by regulating proliferation, differentiation and apoptosis. Evidence from the last few decades supports the idea that accumulated exposure to steroid hormones (for example in post-menopausal women under hormonal replacement therapy) is also a risk factor for breast cancer [5]. The interplay between steroid hormones and radiation-induced risks has been described. For example, we have shown that progesterone protects cultured mammary cells against radiation-induced apoptosis and increases the number of proliferating cells containing chromosomal damage [6]. However, our knowledge of hormonal action in the irradiated breast is far for complete and new discoveries are challenging some established paradigms.

Recently, a lot of attention has be given to a small population of malignant cells thought to be responsible for tumor maintenance and initiation of relapse. These cancer stem cells (CSCs) possess the ability to self-renew (thus to form tumors) and to cause the different lineage of cancer cells comprising a tumor [7]. Breast CSCs were first observed by Al Hajj et al., who described the existence of a subpopulation of CD44^+^CD24^low^ESA^+^lineage^−^ human breast cancer cells capable of initiating tumors in immune-deficient NOD/SCID mice [8]. CSC populations have been defined using several combinations of cell-surface markers, such as CD44^+^CD24^−^ [9], [10], or by measuring cellular activities, such as the expression of aldehyde dehydrogenase (ALDH) [11]. In a recent study, it was shown that breast cancer cell lines contain breast CSCs [12]. CSCs may arise from normal stem cells, or from a differentiated progenitor, which acquired self-renewal abilities. CSCs are thought to be radio-resistant [13], [14] and have a distinct molecular signature [12].

Both estrogens and progesterone have strong proliferative effects on stem/progenitor cells. Several studies have shown that progesterone regulates genes (Notch pathway genes DLL-1, DLL-3, IL6, PRSS2, Interleukins IL6 and IL8 and others) potentially involved in stem cell regulation [15]. Estrogen was recently shown to stimulate CSC expansion through FGF signaling [16]. It was also shown that radiation exposure or steroid hormones can contribute to the initiation of epithelial-to-mesenchymal transition (EMT) and the expansion of CSCs subpopulation [17]. However, to date, the potential involvement of steroid hormones in the radiation-triggered EMT is unknown.

New developments also bring new light into the molecular mechanisms of hormonal action. In the normal human breast, estrogen and progesterone receptors (ER and PR, respectively) are expressed in only 15 to 30% of the luminal epithelial cells and not in other cell types [18]. It is thought that receptor-containing cells secrete paracrine factors that influence the proliferation and activity of nearby receptor-negative cells [19]. Recent investigations have shown that cultured MCF10A normal epithelial cells that do not express PR are nonetheless responsive to progesterone [20]. Furthermore, CSCs can be generated during the transformation of MCF10A cells [21].

In this study, we tested the hypothesis that steroid hormones (estrogen and progesterone) could influence the radiosensitivity of human breast cells and the potential breast cancer risks by stimulating the expansion of breast CSCs. We also evaluated the ability of progesterone to generate CSCs in irradiated PR^−^ MCF10A cells and we measured progesterone-associated miRNA regulations.

## Materials and Methods

### Cell Cultures

T-47D and MCF10A cell lines were provided by Dr Daino (NIRS), MCF7 cell line was provided by Dr Mori (NIRS). T-47D and MCF7 breast cancer cell lines were maintained as previously [6]in Dulbecco’s modified Eagle medium (DMEM) with 4.5 g/L glucose, 0.11 g/L sodium pyruvate, glutamate (GlutaMAX 1t) and pyridoxine, supplemented with 5% fetal calf serum, penicillin and streptomycin. Non-tumorigenic MCF10A breast epithelial cells [22] were maintained in DMEM/F12 supplemented with 5% horse serum, 20 ng/mL epidermal growth factor (EGF), 10 µg/mL insulin, 100 µg/mL hydrocortisone and 10 ng/mL cholera toxin. Cultures were grown in 5% CO_2_ at 95% humidity.

### Irradiation and Hormonal Treatment

Cells were irradiated in serum-free medium 72 hours after plating, using an X-ray generator (ISOVOLT Titan-320, General Electric, Fairfield, CT, USA). Irradiation dose was 10 Gy at a dose-rate of 0.9 Gy/min. Starting one day after plating, natural progesterone and estrogen diluted in ethanol (10 µM) were added to the culture medium once a day until cell collection at a final concentration of 10 nM. MCF10A cells were also cotreated with 10 µM mifepristone (diluted in ethanol) or 10 µM PD173074 inhibitor (diluted in DMSO).

### Proliferation and Survival

At least 200 cells per sample (in three separate experiments) were scored for proliferation and survival analysis. Discrimination between viable and dead cells (including dead cells in the supernatant) was performed after trypan blue staining.

### Measurement of ROS Levels

Intracellular levels of ROS in MCF10A cells were measured using 5-(and-6)-chloromethyl-2′,7′-dichlorodihydrofluorescein diacetate, acetyl ester (CM-H2DCFDA, Molecular Probes, Eugene, OR, USA). Cells were plated in 12-well plates and loaded with pre-warmed PBS containing 10 µM CM-H2DCFDA. Next, cells were returned to pre-warmed medium and incubated for 40 minutes with 10 nM progesterone and mifepristone. Then, fluorescence intensities were measured using a SpectraMax M5 microplate reader (Molecular Devices, Sunnyvale, CA, USA) (excitation at 493 nm, emission at 520 nm). Unstained cells were used as negative control. Cells treated with 100 µM H2O2 were used as positive control.

### Separation of ALDEFLUOR-positive Cells by Flow Cytometry

ALDH activity in the cells was evaluated by flow cytometry using the ALDEFLUOR kit (Stemcell technologies, Vancouver, BC, Canada). Cells expressing with low and high levels of ALDH enzymatic activity (respectively ALDH^−^ and ALDH^+^ cells) were identified and sorted with a FACSAria cell sorter (BD Biosciences, Franklin Lakes, NJ, USA). As a negative control, cells were treated with diethylaminobenzaldehyde, a specific ALDH inhibitor.

### Anchorage-independent Culture

Sorted ALDH^−^ and ALDH^+^ cells were resuspended in complete mammosphere cell culture medium (MammoCult; Stemcell Technologies) supplemented with Mammocult proliferation supplement, hydrocortisone and heparin. Then they were seeded in ultra-low adherent plates (Corning, Corning, NY, USA) at densities of 5,000 to 40,000 cells per well and grown for 7 days. Spheres larger than 60 µm in size were counted.

### Total RNA Extraction

Total RNA containing microRNAs was extracted using TRIzol and a protocol slightly modified from the manufacturer’s instructions. During the precipitation phase, 0.8 mL of isopropanol was added per 1 mL of TRIzol reagent, then the samples were incubated for 2–3 min at room temperature. RNA was washed with 70% ethanol. Quantity and quality of RNA samples was evaluated using a NanoDrop ND-1000 spectrophotometer (NanoDrop Technologies, Montchanin, DE, USA).

### Real-time PCR-based miRNA Expression Profiling

250 ng RNA per sample was reverse transcribed using the RT^2^ first strand kit (SABiosciences, Frederick, MD, USA), then real-time PCR reactions were performed in triplicate with an Applied Biosystems 7300 Real-Time PCR system (Life Technologies, Carlsbad, CA, USA), using the RT2 SYBR Green PCR Master Mix (SABiosciences) on 96-well Human Breast Cancer miRNA PCR Arrays (MIHS-109Z, SABiosciences), which allowed to analyze the differential expression of 84 miRNAs known or predicted to be associated with breast cancer, according to the manufacturer’s instructions.

### microRNA Expression Data Analysis

Data analysis was performed using the web-based miRNA PCR Data Analysis Software from SABiosciences (http://pcrdataanalysis.sabiosciences.com/mirna/arrayanalysis.php). The ΔΔCt_2_ method was used the relative microRNA expression levels in each group. For each microRNA, fold changes (compared to levels in control cells) were calculated, then expressed as fold regulations (for fold changes <1, fold regulations were equal to −1/fold change; for fold changes ≥1, fold regulations were equal to fold change).

We identified the molecular pathways potentially altered by deregulated microRNAs (fold change >4, p<0.05) using the DIANA-mirPath software combined with the DIANA-microT v3.0 prediction software [23], which provided a list of enriched KEGG pathways, with associated p-values.

## Results

### Steroid Hormones Modulate Radiation-induced Cell Death

**Figure 1:**
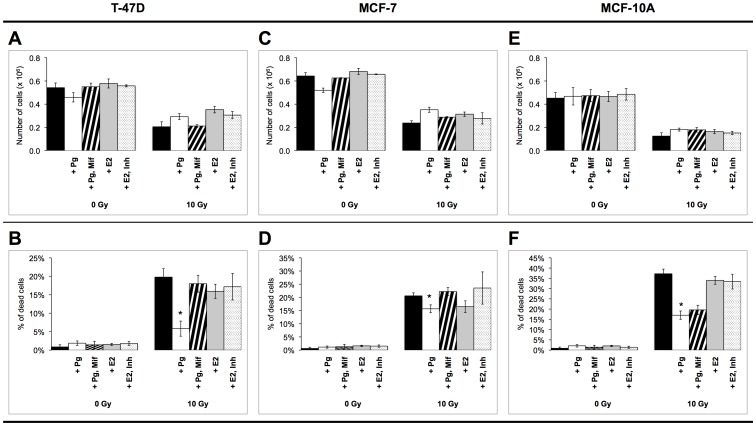
Proliferation and viability of MCF7, T47D and MCF10A cells after irradiation and steroid hormone treatment. Proliferation of T47D (A), MCF7 (C) and MCF10A (E) cells and the number of dead T47D cells (B), MCF7 (D) and MCF10A (F) cells were measured three days after irradiation. Cells were seeded at a density of 10^5^ cells/mL three days before irradiation. Hormonal treatment was performed two days before irradiation and every day afterwards. *Pg*: progesterone, *Mif*: mifespristone, *E2*: estrogen, *Inh*: PD173074. Results are representative of at least three independent experiments. Error bars represent standard deviation. Asterisks denote significant differences (*t-*test, *p<0.05, **p<0.01).

We first measured proliferation and viability three days after X-ray irradiation in cultured steroid receptor-positive and receptor-negative cells exposed to progesterone and estrogens treatment (Fig. 1). In order to assess the role of PR in the observed progesterone-induced effects, cells were also treated with mifepristone, a PR antagonist. Because estrogen effects were shown to be mediated through FGF signaling, cells were treated as well with PD173074, an FGF-receptor inhibitor.

Exposure to 10 Gy X-rays inhibited proliferation in T47D, MCF7 and MCF10A cells (Fig. 1A, C, E). Exposure to progesterone inhibited the proliferation of non-irradiated T47D and MCF7 cells, but did not modulate the proliferation of MCF10A cells. Progesterone stimulated the proliferation of irradiated T47D, MCF7 and MCF10A cells. Progesterone effects on the proliferation of T47D and MCF7 cells were inhibited by mifepristone, suggesting that progesterone mediated its effects through PR. Mifepristone did not have any effect on the proliferation of MCF10A non-irradiated and irradiated cells, indicating that progesterone effect on MCF10A proliferation did not rely on PR.

The percentage of dead cells was not significantly modulated by hormonal treatment (it did not exceed 2% in each cell line), but it increased significantly after exposure to 10 Gy X-rays (Fig. 1B, D, F). In accordance with our earlier results [6], progesterone treatment significantly reduced the radiation-induced cell death in T47D cells. Interestingly, a similar protective effect of progesterone was also observed in MCF10A cells. Co-treatment with mifepristone counteracted the protective effect of progesterone in T47D but not in MCF10A cells, confirming that this hormonal protective effect did not rely on PR in MCF10A cells. Even though progesterone did not significantly decrease the percentage of dead MCF7 cells, we observed a significant increase of dead MCF7 cells after co-treatment with mifepristone, which might suggest that progesterone might also slightly protect MCF7 cells against radiation-induced cell death through PR.

On the contrary, estrogen treatment elicited a weak protective effect only in T47D cells (about 15% dead cells), as the percentages of dead cells were non significantly different in treated and non-treated irradiated MCF7 and MCF10A cells (Fig. 1B, D and F). Co-treatment of irradiated MCF7 cells with PD173074 inhibitor drew inconclusive results, because the differences were not significant.

In summary, progesterone and oestrogen partly counteracted the radiation-induced proliferation inhibition. Progesterone protected against radiation-induced cell death. In PR^−^ MCF10A cells, progesterone effects were independent of PR expression.

### Ionizing Radiation and Steroid Hormones Increase the Proportion of ALDH^+^ Cells

**Figure 2:**
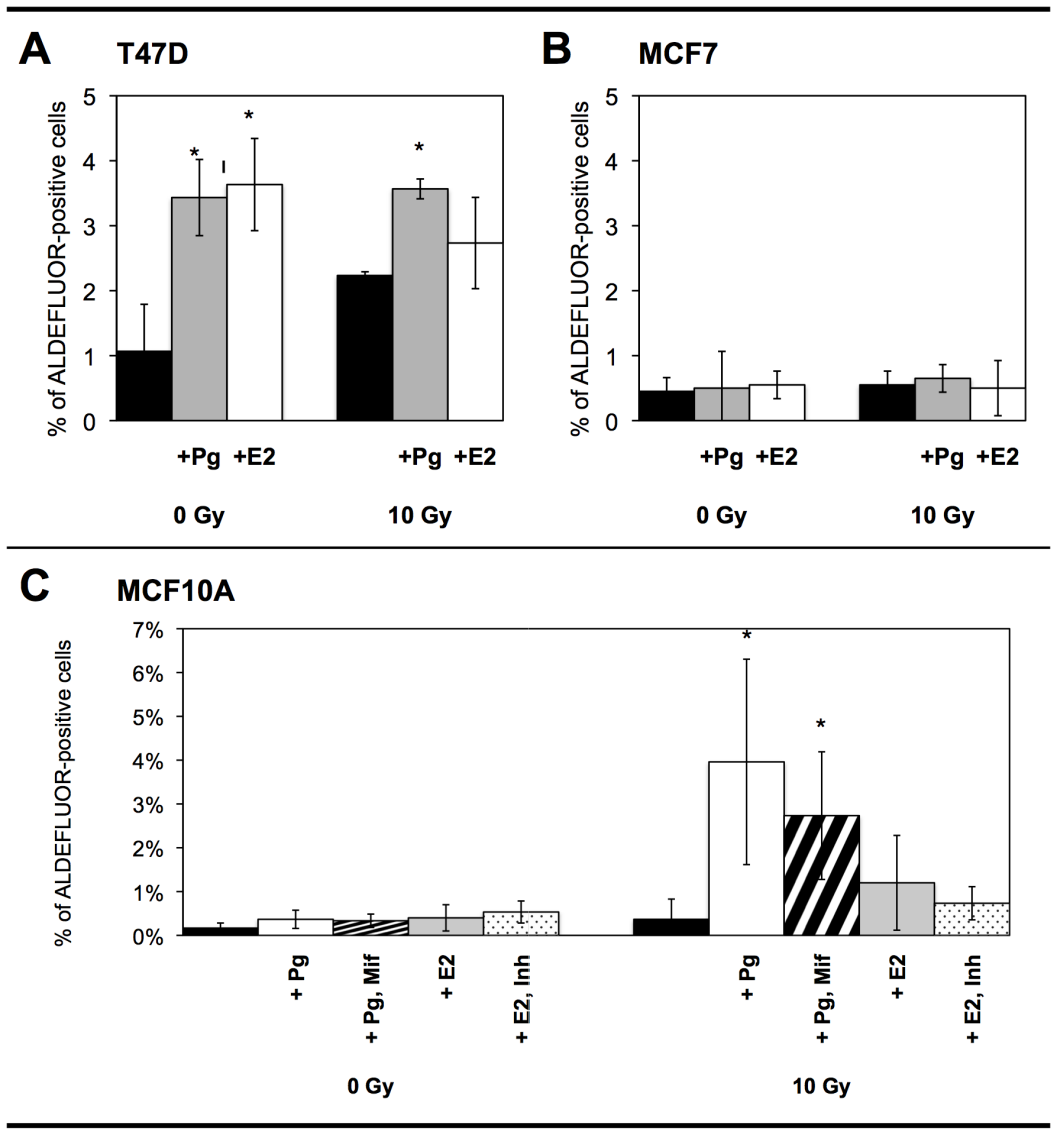
Proportion of CSCs after irradiation and steroid hormone treatment. The percentages of ALDH^+^ T47D (A), MCF7 (B) and MCF10A (C) cells were evaluated by flow cytometry three days after irradiation. Hormonal treatment was performed two days before irradiation and every day afterwards. Pg: progesterone, Mif: mifespristone, E2: estrogen, Inh: PD173074. Results are representative of at least three independent experiments. Error bars represent standard deviation. Asterisks denote significant differences (*t-*test, *p<0.05, **p<0.01).

We measured the proportion of cancer stem cells (CSCs) by flow cytometry, using activity of aldehyde dehydrogenase (ALDH) as a marker with the ALDEFLUOR kit (Fig. 2). About 1% of untreated T47D cells, 0.5% of untreated MCF7 cells and 0.2% of untreated MCF10A cells were ALDH^+^. On the contrary, the proportion of ALDH^+^ T47D cells was significantly increased after 10 Gy irradiation (2.2%) and after hormonal treatment with progesterone (3.4%) and estrogen (3.6%). Progesterone treatment of irradiated T47D cells resulted in a similar increase in the proportion of ALDH^+^ cells (3.6%), but no additive effect of irradiation and progesterone treatment was observed. The proportion of ALDH^+^ cells after estrogen treatment of irradiated T47D cells was slightly higher than in irradiated non-treated cells, but slightly lower than in treated non-irradiated cells (2.7%, non-significant differences). In the MCF10A cell line, no increase in the proportion of ALDH^+^ cells was observed after hormonal treatment or after 10 Gy radiation exposure alone (less than 0.5%). A significant increase was observed only when MCF10A cells were exposed both to progesterone treatment and radiation exposure (4.0%). Cotreatment with mifepristone did not significantly reduce the proportion of ALDH^+^ cells (2.7%). Significant increases in the proportion of CD44+/CD24- cells were also observed after progesterone treatment and radiation exposure (Figure S1). These results suggested that ionizing radiation and/or steroid hormone treatment could stimulate the expansion of CSCs. In irradiated MCF10A cells, progesterone action was independent of PR expression.

### ALDH^+^ Cells Show Increased Tumorphere-forming Abilities and Radioresistance

**Figure 3:**
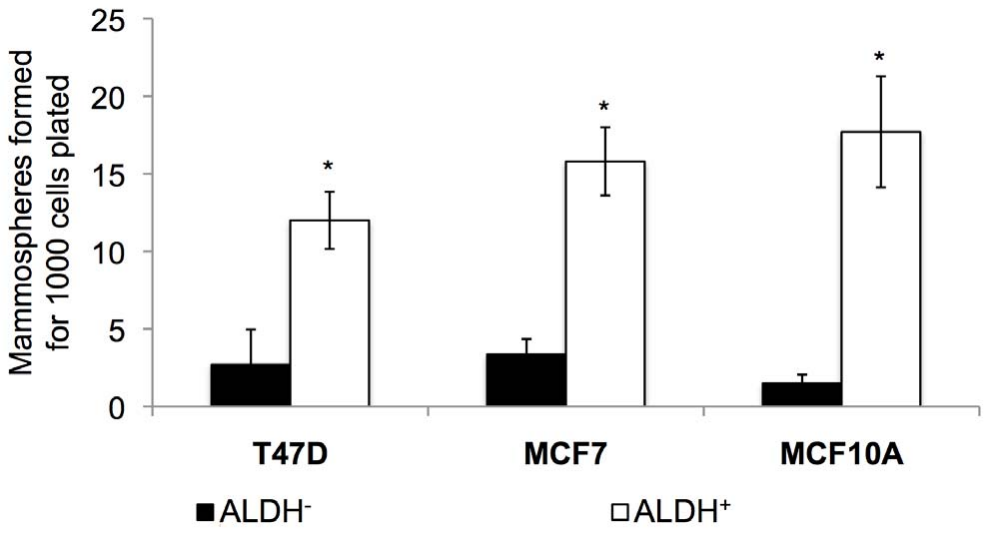
Mammosphere-forming ability of ALDH^−^ cells and ALDH^+^ CSCs. Sorted ALDH^−^ and ALDH^+^ T47D, MCF7 and MCF10A cells were plated in ultra-low adherence plates and the number of mammospheres formed after 7 days was counted. Error bars represent standard deviation. Results are representative of three independent experiments. Error bars represent standard deviation. Asterisks denote significant differences (*t-*test, *p<0.05, **p<0.01).

In order to confirm whether the population of ALDH^+^ cells had indeed tumor-initiating ability, we measured the capacity of ALDH^+^ cells to grow “tumorspheres” (or “mammospheres”) in anchorage-independent conditions [12]. In each cell line, ALDH^+^ cells showed increased tumorsphere-forming capacity, compared with ALDH^−^ cells, as observed in Fig. 3 (the numbers of mammospheres formed for 1000 cells plated were: T47D: 2.7 ALDH^−^, 12 ALDH^+^/MCF7∶3.4 ALDH^−^, 15.8 ALDH^+^/MCF10A: 1.5 ALDH^−^, 17.7 ALDH^+^).

**Figure 4:**
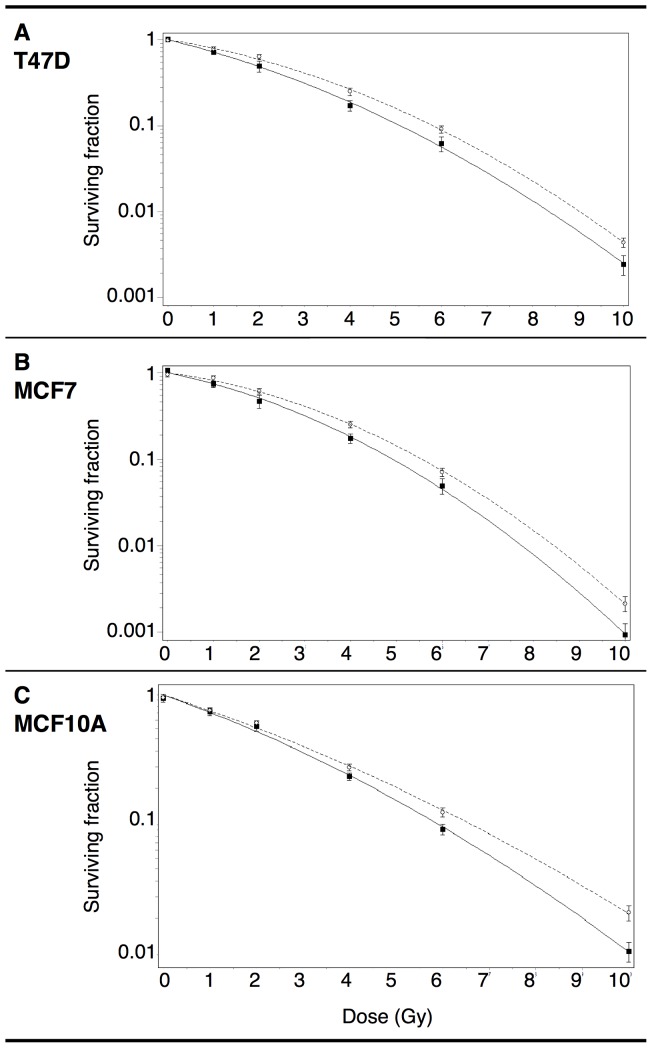
Dose-response curves for clonogenic survival of ALDH^−^ cells and ALDH^+^ CSCs. Sorted ALDH^−^ (squares, continuous lines) and ALDH^+^ (circles, dotted lines) T47D (A), MCF7 (B) and MCF10A (C) cells were exposed to various doses of ionizing radiation. Lines represented fitted curves according to linear quadratic regression. Results are representative of at least three independent experiments. Error bars represent standard deviation. Statistical significance of the difference between dose-response curves (p<0.05) was performed using one-way Analysis of Variance (one-way ANOVA) with Bonferroni correction for pairwise group comparisons.

Reproductive clonogenic viability of ALDH^−^ and ALDH^+^ cells was evaluated by the colony forming assay (Fig. 4). In each cell line, ALDH^+^ cells showed increased radioresistance compared to ALDH^−^ cells. The dose that gave 50% mean clonogenic survival was higher in ALDH^+^ (T47D: 2.5 Gy/MCF7∶2.5 Gy/MCF10A: 2.3 Gy) than in ALDH^−^ cells (T47D: 1.9 Gy/MCF7∶2 Gy/MCF10A: 2.1 Gy).

Overall these results suggested that the ALDH+ cell population was enriched in radioresistant CSCs.

### Progesterone Treatment in MCF10A Cells Results in Increased ROS Levels

**Figure 5:**
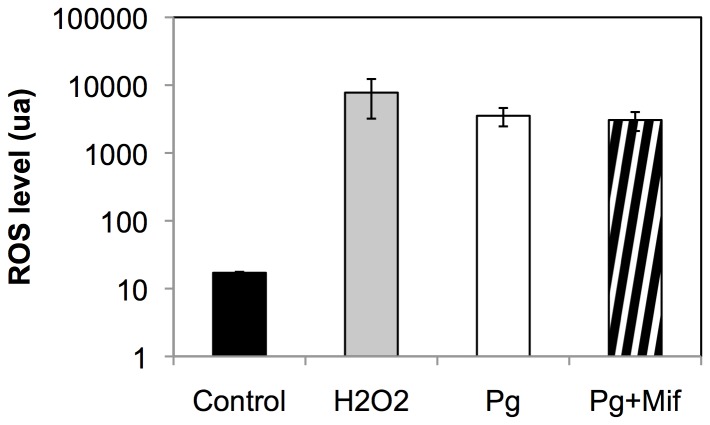
ROS levels in MCF10A cells. ROS levels were measured 40 minutes after treatment with progesterone or co-treatment with progesterone and mifepristone. As a positive control, MCF10A were treated with H2O2. Results are representative of at least three independent experiments. Error bars represent standard deviation. Asterisks denote significant differences (*t-*test, **p<0.01).

Because the observed progesterone effects in MCF10A cells were independent of PR expression, we decided to assess its possible mechanisms of action. In order to confirm recent reports of non-genomic action of progesterone in MCF10A cells, we first measured ROS levels after progesterone treatment in MCF10A cells. 40 minutes after progesterone addition, ROS levels were significantly higher than in control cells (Fig. 5). Co-treatment with mifepristone did not decrease the ROS levels.

### The Modulation of CSC Levels by Progesterone and Radiation in MCF10A Cells Involves Cancer-associated microRNA Regulations

**Figure 6:**
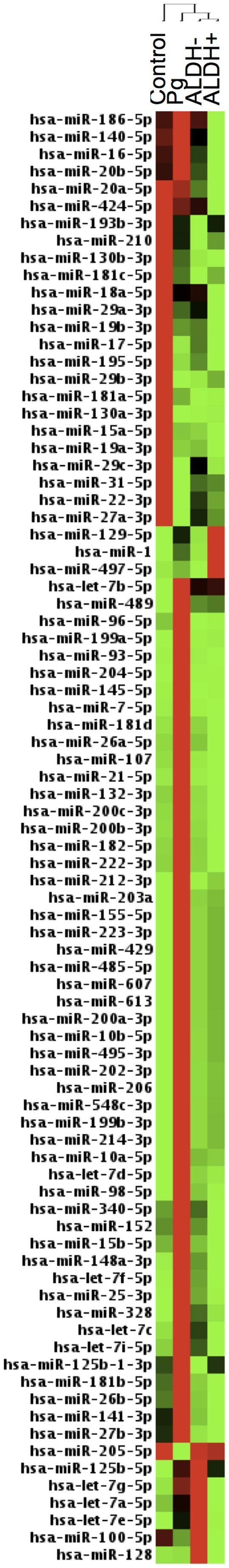
Heatmap of microRNA expression in MCF10A cells. Each column represents one experimental group and each row represents one microRNA. microRNAs were arranged by unsupervised hierarchical clustering. Green and red indicate down- and upregulation, respectively, relative to the overall mean expression for each microRNA. The four experimental groups were: 1) non-irradiated cells (*control*); 2) cells exposed to progesterone treatment alone (*Pg*); 3) ALDH− cells and 4) ALDH+ cells after irradiation and progesterone treatment.

**Figure 7:**
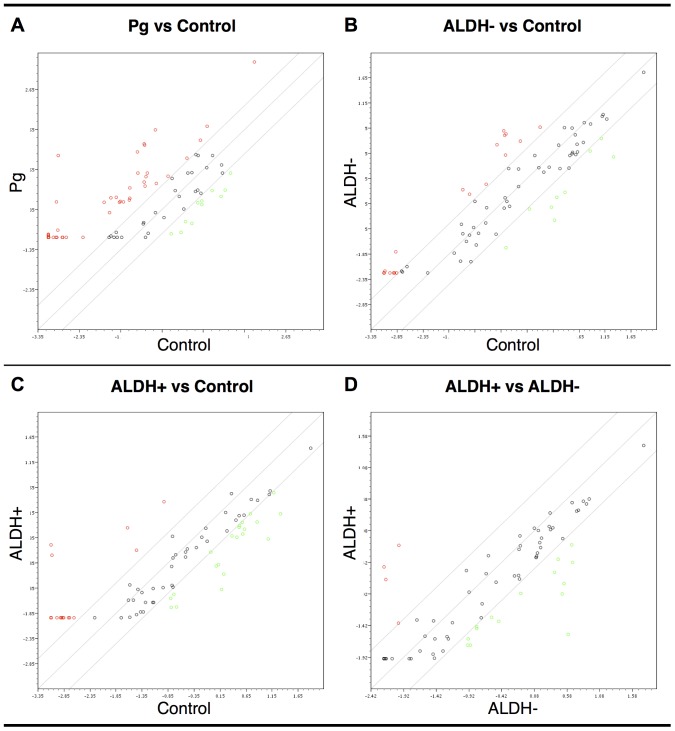
Comparative microRNA expression levels in MCF10A cells. Each scatter plot shows microRNA expression levels (logarithmic scale) for two experimental groups: cells exposed to progesterone treatment alone (*Pg*) vs non-irradiated cells (*Control*) (A), ALDH− cells (after irradiation and progesterone treatment) vs *Control* (B), ALDH+ cells (after irradiation and progesterone treatment) vs *Control* (C), ALDH+ cells vs ALDH− cells (D). The outer diagonal lines indicate 4-fold differences in microRNA expression. Each microRNA is represented by a circle.

In order to assess whether progesterone action in MCF10A cells involved miRNA regulations, we measured using PCR arrays the expression levels of 84 miRNAs known or predicted to be regulated during breast cancer initiation or progression. miRNA levels were profiled in (1) untreated control MCF10A cells, (2) MCF10A cells exposed to progesterone treatment alone, (3) ALDH^−^ cells and (4) ALDH^+^ cells after progesterone treatment and irradiation. Fig. 6 shows a heatmap of microRNA expression levels for each group. Fold changes compared to control group were calculated and expressed as fold regulations. Comparative microRNA expression levels are presented in Fig. 7. Some significant microRNA regulations (fold change >4 or <−4, p<0.05) are presented in Table 1. A functional analysis of the gene targets of these microRNAs was performed using the DIANA-miRPath software. An enrichment analysis of these gene targets provided a list of cell functions and pathways (based on Kyoto Enyclopedia of Genes and Genomes – KEGG – nomenclature) likely to be affected or involved in each experimental group (Table 2, p<0.01). Several of these KEGG pathways were related to cellular interactions (*ECM–receptor interaction, Focal adhesion, Adherens junction*), signaling pathways (*MAPK signaling pathway, p53 signaling pathway, VEGF signaling pathway, Phosphatidylinositol signaling system, TGF-beta signaling pathway*) or other cancers. A similar enrichment analysis was performed to compare target gene functions in ALDH^−^ and ALDH^+^ cells (Table 3); these functions were related to cell adherence (*ECM –receptor interaction, Focal adhesion, Adherens junction*), signaling pathways (*MAPK signaling pathway, TGF-beta signaling pathway*) and other cancers.

**Table 1:** Significant microRNA regulations in MCF10A cells.

	miRNA	Fold regulation	p-value
**Pg vs Control**	miR-22-3p	−11.0	0.01
	miR-29c-3p	−3.5	0.03
	miR-328	33.9	0.00
	miR-98-5p	15.4	0.03
**ALDH− vs Control**	miR-19a-3p	−4.7	0.01
	miR-210	−9.3	0.03
	miR-29b-3p	−10.0	0.03
	Let-7e-5p	19.7	0.04
**ALDH+ vs Control**	miR-128	−5.7	0.03
	miR-15b-5p	−4.2	0.03
	miR-17-5p	−6.3	0.02
	miR-195-5p	−28.4	0.04
	miR-19a-3p	−8.0	0.00
	miR-20b-5p	−4.0	0.04
	miR-22-3p	−4.3	0.03
**ALDH+ vs ALDH−**	Let-7e-5p	−19.6	0.04
	Let-7f-5p	−26.6	0.03

MicroRNA fold regulations were expressed relative to non-irradiated cells (*Control*) or to ALDH− cells. MicroRNAs regulated more than 4-fold (p<0.05) were reported.

**Table 2:** Functional analysis of the genes targeted by microRNA regulations.

Progesterone		ALDH−		ALDH^+^	
KEGG pathways	No of genes	KEGG pathways	No of genes	KEGG pathways	No of genes
ECM-receptor interaction	26	Focal adhesion	19	Axon guidance	10
Focal adhesion	45	ECM-receptor interaction	12	Renal cell carcinoma	7
MAPK signaling pathway	49	Cell Communication	12	TGF-beta signaling pathway	8
p53 signaling pathway	18	mTOR signaling pathway	7	Pancreatic cancer	7
Glioma	17	Small cell lung cancer	7	Chronic myeloid leukemia	7
Oxidative phosphorylation	1	Prostate cancer	7	Bladder cancer	5
Amyotrophic lateral sclerosis	7	Melanoma	6	Wnt signaling pathway	10
Melanoma	17	Glioma	5	p53 signaling pathway	6
Adherens junction	17	VEGF signaling pathway	5	Prostate cancer	7
Glycerophospholipid metabolism	16	Endometrial cancer	4	Focal adhesion	11
Small cell lung cancer	19	T cell receptor signaling pathway	5	MAPK signaling pathway	13
Regulation of actin cytoskeleton	37	B cell receptor signaling pathway	4	GnRH signaling pathway	7
Glycan structures - biosynthesis 1	24	Renal cell carcinoma	4	Colorectal cancer	6
Ribosome	1	Type II diabetes mellitus	3		
T cell receptor signaling pathway	19	C5-Branched dibasic acid metabolism	1		
Colorectal cancer	18	Phosphatidylinositol signaling system	4		
		Pancreatic cancer	4		
		Apoptosis	4		
		Heparan sulfate biosynthesis	2		
		Insulin signaling pathway	5		
		Non-small cell lung cancer	3		
		Acute myeloid leukemia	3		

Analysis of the gene targeted by each significantly modulated microRNA in each experimental group (relative to control) revealed several significantly enriched KEGG pathways (p<0.01). KEGG pathway names and the number of associated target genes are reported in this table.

**Table 3:** Functional analysis of the genes targeted by microRNA regulations in CSCs.

ALDH+ vs ALDH−	No of genes
TGF-beta signaling pathway	9
MAPK signaling pathway	16
Colorectal cancer	8
Adherens junction	7
Type II diabetes mellitus	5
Pancreatic cancer	6
Chronic myeloid leukemia	6
ECM-receptor interaction	6
Focal adhesion	10

Analysis of the gene targeted by each significantly modulated microRNA in ALDH+ cells, relative to ALDH− cells, revealed several significantly enriched KEGG pathways (p<0.01). KEGG pathway names and the number of associated target genes are reported in this table.

## Discussion

In the light of increased breast cancer risks in women exposed to ionizing radiation, it is crucial to better evaluate the various additional risk factors that could further result in breast cancer. There is strong evidence that steroid hormones influence breast cancer risk, but the molecular mechanisms are poorly understood. We, and others, had previously shown that steroid hormones can influence radiosensitivity in breast cells. In this study, we assessed *in vitro* the potential role of breast cancer stem cells (CSCs) in the modulation of radiosensitivity by steroid hormones, and we observed for the first time that progesterone treatment of irradiated PR- cells results in increased numbers of CSCs.

A protective effect of progesterone (in the three cell lines) and estrogen (in MCF7 cells only) against radiation-induced cell death was observed, confirming earlier reports [24]–[26]. Unsurprisingly, the protective effect of progesterone was dependent on PR in T47D and MCF7 cells, but not in MCF10A cells. This protective effect was a direct effect of progesterone on the induction of radiation-induced apoptosis, which might result from the modulation of pro- or anti-apoptotic genes, such as HRK [6], or from the regulation of cell signaling pathways involved in apoptosis (PI3/Akt…). In MCF10A cells, it was shown that progesterone treatment inhibited apoptosis induced by activation of the FasL pathway, as seen by decreased caspase 3 and caspase 7 levels [20]. The apoptosis-regulating properties of estrogen are also well known: depending on the experimental model, estrogen action has been described as anti-apoptotic or pro-apoptotic [27]; in T47D and MCF7 cells, estrogen regulates the expression of anti-apoptotic proteins such as bcl-2 [28]. However, only a weak protective effect of estrogen was observed in our study.

Compared to their non-CSC counterparts, CSCs in each cell line showed increased radioresistance, in accordance with published data. For example, in MCF7 and MDA-MB-231 cell lines, CSCs (CD44^+^/CD24^−^) were shown to be more radioresistant than non-CSCs, based on clonogenic survival, ROS levels and phosphorylation of γH2AX [29]. Radioresistance of progenitors cells was also shown in several other models. For example, mouse mammary stem cells (defined as a lin^−^CD24^+^CD29^+^ side population) exhibited resistance to radiation [30]. Radiation exposure resulted in the expansion of human (MCF7) and murine side population progenitors [30].

We measured the proportion of CSCs in the three cell lines in order to assess the role of CSCs in this hormonal modulation of radiosensitivity. Neither hormonal treatment nor irradiation modulated the proportion of ALDH^+^ MCF7 cells. We did not observe any increase in the proportion of ALDH^+^ MCF7 cells after estrogen treatment, contrary to a recent report showing an expansion of CD44+/CD24− cells [16]. However, estrogen was added at a final concentration of 10 nM in our study and 1 nM in that other study; a possible dose effect cannot be ruled out. On the contrary, progesterone and estrogen stimulated the expansion of the CSC population in irradiated and non-irradiated luminal breast cancer T47D cells, which could result either from a stimulation of CSC proliferation or from the reprogramming of non-CSCs which would acquire a stem-like phenotype. In the normal mammary gland, stem/progenitor cells do not express progesterone receptor (PR) nor estrogen receptor (ER), but receive hormonal paracrine signaling from luminal PR^+^/ER^+^ cells [31]. Similarly, in cultured breast cancer cells, the non-CSC compartment might stimulate the expansion of CSCs through paracrine signaling. Indeed, it has been shown that the secretion of FGF9 in estrogen-treated MCF7 cells leads to the increase of the CSC population [16]. On the other hand, in T47D cells, progesterone was shown to transform PR^+^/ER^+^ cells into PR^−/^ER^−^ cells expressing a myoepithelial CK5^+^ phenotype (associated with stemness in the human breast), through an autocrine mechanism [15], [32], suggesting that progesterone might contribute to the transformation of cells into CSCs.

It is generally accepted that in the normal breast, steroid hormones target only a small proportion of hormone receptor-expressing cells, which communicate with other cells through paracrine interactions [7]. However, some evidence has emerged showing that PR-negative MCF10A cells are also directly responsive to progesterone [20], [33], [34], suggesting that progesterone action in the normal breast might also target PR-negative epithelial cells. For this reason, we investigated whether progesterone could influence the proportion of CSCs in irradiated MCF10A cells. Untreated MCF10A containted less than 0.5% CSCs [12]. Progesterone treatment of non-irradiated MCF10A did not trigger any effect at any of the considered endpoints (proliferation, cell death, proportion of CSCs), but the fact that we observed hormonal effects (protection against radiation-induced cell death and increased numbers of CSCs) in irradiated cells suggested that progesterone nonetheless has direct action on MCF10A cells.

To date, our understanding of non PR-related progesterone action remains partial. Several controversial novel candidate receptors mediating genomic and non-genomic progesterone effects were identified during the last decade. A G-protein coupled receptor, called membrane (m)-PR, was first characterized in fish ovaries [35]. The three human mPR isoforms (mPRα, mPRβ and mPRχ) present different tissue distributions and expression patterns through the reproductive cycle. mPRα expression was observed in MCF7 and SK-BR-3 cells, and its expression was higher in breast tumor biopsies than in normal tissue from the same breast [36]. However, the role of mPRs in progesterone signaling is debated by several studies questioning its localization or even its ability to bind progesterone [37], [38]. Progesterone membrane receptor component 1 (PGMRC1) is another membrane progesterone receptor [39], whose expression was observed in various models including breast cancer cells.

In MCF10A cells, non-genomic action of progesterone resulted in increased mitochondrial activity (observed as increased mitochondrial potential) and subsequent inhibition of Fas-induced apoptosis [20]. A strong correlation exists between mitochondrial membrane potential and reactive oxygen species (ROS) levels [40], [41]. In accordance with these results, we observed a PR-independent increase in ROS levels after progesterone treatment of MCF10A cells.

In addition to non-genomic effects, our results indicated that progesterone action in MCF10A cells involves genomic effects. Several microRNAs were strongly down- (miR-22-3p, miR-29c-3p) or upregulated (miR-328, miR-98-5p) by progesterone and might contribute to the observed hormonal effects. miR-22 is a tumor suppressor that induces cellular senescence (by targeting CDK6, SIRT1 and Sp1) [42] and is frequently downregulated in ER^+^ breast cancer [43]. miR-29c is downregulated in inflammatory breast cancer [44] and its expression is associated with good prognosis [45]. Another recent study has shown that progesterone decreased miR-29 expression in breast cancer cells lines expressing PR and ER (T47D and BT474), resulting in the upregulation of Krüppel-like factor 4 (KLF4), a transcription factor required for the dedifferentiation into pluripotent stem cell phenotype and for the maintenance of CSCs; as a consequence, the authors observed an expansion of CK5^+^/CD44^+^ tumor-initiating cells [46]. Compared to control cells, both miR-22 and miR-29c were downregulated not only after progesterone treatment, but also after irradiation in ADLH^−^ and ALDH^+^ cells. miR-98, a Let-7 family member, was recently shown to inhibit Fas-mediated apoptosis in Hela cells [47], in accordance with the anti-apoptotic effect of progesterone. The precise role of miR-238 in human cancer is not yet fully elucidated: while it was overexpressed in patients with non-small cell lung cancer brain [48], loss of miR-328 expression occurred in blast crisis chronic myelogenous leukemia [49]. The involvement of miR-328 in breast cancer has not been established to date. We performed a functional analysis of the target genes for the microRNAs deregulated after progesterone treatment and irradiation. The modulation of cell adhesion (*ECM-receptor interaction, Focal adhesion…*) and the remodeling of actin cytoskeleton are functions which might be important for epithelial-to-mesenchymal transition (EMT) and cancer initiation [50]. Ionizing radiation was previously shown to predispose breast cells to transforming growth factor beta (TGFβ)-induced EMT [17], independently of radiation dose or LET [51]. Overall the observed microRNA regulations are consistent with cancer-related processes.

Control and progesterone-treated MCF10A populations contained negligible amounts of CSCs. On the contrary, after irradiation and progesterone treatment, the cell population contained both non-CSCs and CSCs, whose microRNA expression patterns were different both from each other and from non-irradiated cells (control and progesterone-treated cells). Using an inducible breast oncogenesis model based on MCF10A cells, Iliopoulos *et al.* have shown that CSCs and non-CSCs exist in a dynamic equilibrium that can be influenced by an inflammatory feedback loop involving NF-κB, Lin28, IL6, STAT3, PTEN, CYLD and several microRNAs (let-7, miR-21, miR-181b-1) [21]. In our study, after irradiation and progesterone treatment, two let-7 family microRNAs were significantly downregulated in ALDH^+^ cells, compared to ALDH^−^ cells (Table 1). The let-7 family microRNAs (which includes 13 human homologues) were among the first to be directly described as tumor suppressors, by negatively regulating the expression of the Ras oncogene [52]. Loss of let-7 expression was observed in many human cancers and is associated with poor survival [53] and stem cell phenotype [54].

Taken together, our results suggested that the increased numbers of CSCs induced by steroid hormones might contribute to the modulation of radiosensitivity by the hormone in T47D breast cancer cells, but not in MCF10A cells. However, we report for the first time that progesterone directly triggered microRNA regulations and modulated the radiosensitivity of normal breast epithelial cells lacking the expression of PR, suggesting that the classical model of hormonal paracrine action in the normal breast [55] may need to be completed. Furthermore, the combination of progesterone treatment and radiation exposure was capable of generating CSCs. The origin of CSCs is still a matter of ample controversy: some have suggested that CSCs might result from already malignant cells through a clonal evolution process [56]. Our results are consistent with the idea that progesterone and radiation exposure might trigger or contribute to cancer initiation events, resulting in the appearance of CSCs.

Although MCF10A possess some genetic abnormalities, they are generally considered as a “normal” cell line, whose morphogenesis on reconstituted basement membrane is similar to what is observed with normal breast epithelial cells [57]. MCF10A cells express markers associated with a basal phenotype [58], which can give rise to basal-type cancers. On that account, MCF10A might be an appropriate model for evaluating breast cancer risks and initiation. Therefore, we can hypothesize that the combined effects of irradiation and progesterone on tumor-initiating CSCs might contribute to additional cancer risk [59] in the normal breast.

Additionally, increased numbers of CSCs were also observed in PR^+^/ER^+^ T47D breast cancer cells after steroid hormone treatment and irradiation. Convincing evidence suggest a link between CSCs and metastasis in cancer models [60]. For example, metastatis in inflammatory breast cancer is mediated by ALDH^+^ CSCs [61]. Similarly, human CSCs are involved in spontaneous metastasis in mouse xenographt tumor models [62]. Therefore, expansion of CSCs by progesterone in breast cancer might result in additional metastasis risk.

In conclusion, our results suggest that progesterone might influence radiation-induced breast cancer risk by generating tumor-initiating breast cancer stem cells. In order to decrease the potential risks of breast cancer resulting from chest ionizing radiation exposure, it might be useful to take into account the variability of progesterone levels during menstrual cycle and between individuals. Our results also shed additional light on elevated breast cancer risks in women treated with hormone replacement therapy [63]. Further investigations are needed to better understand the mechanisms involved in PR-independent progesterone action in the normal breast and the generation of CSCs after exposure to ionizing radiation, in particular in the low-dose range.

## Supporting Information

Figure S1 **Proportion of CD44+/CD24− CSCs after irradiation and steroid hormone treatment.** The percentages of CD44^+^/CD24^−^ MCF10A cells were evaluated by flow cytometry three days after irradiation, after labeling with conjugated anti-human CD133−PE (phycoerythrin; Miltenyi Biotec) and CD44−FITC (Miltenyi Biotec). Hormonal treatment was performed two days before irradiation and every day afterwards. Pg: progesterone. Results are representative of three independent experiments. Error bars represent standard deviation. Asterisks denote significant differences (t-test, *p<0.05).
(TIFF)

Table S1 **microRNA expression levels in MCF10A cells, compared to control.** For each experimental group (cells exposed to progesterone treatment alone, ALDH− cells and ALDH+ cells after irradiation and progesterone treatment), fold-changes (FCs) of miRNA expression were measured as compared to non-irradiated and non-treated control cells. If the expression ratios were >1, then FCs were equal to expression ratios. If the expression ratios were <1, then FCs were equal to the opposite of expression ratios.
(PDF)
