# Supplementary figures and images for: Generation of Breast Cancer Stem Cells by Steroid Hormones in Irradiated Human Mammary Cell Lines
Source: PLoS One. 2013 Oct 16;8(10):e77124. doi: 10.1371/journal.pone.0077124 (PMC3797732; doi:10.1371/journal.pone.0077124)

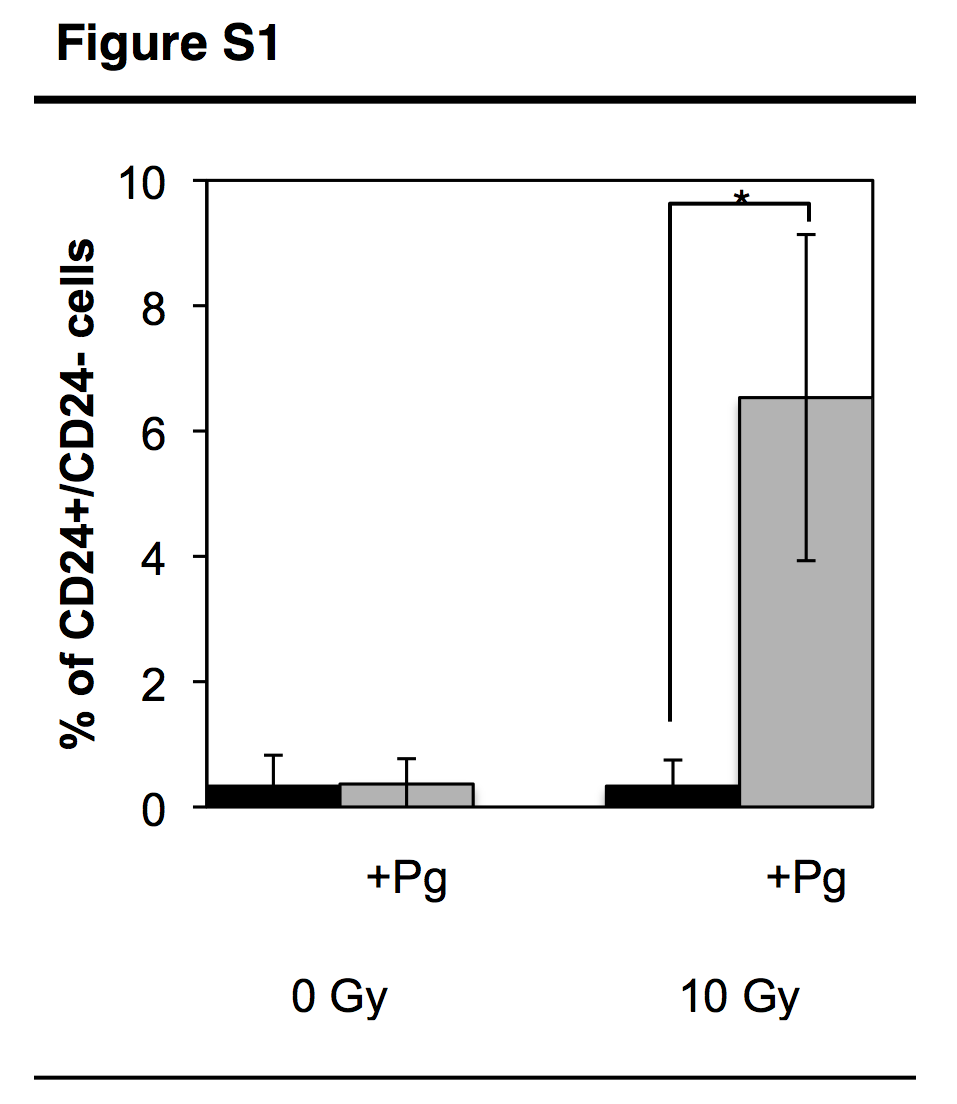

Supplement: Figure S1 — Proportion of CD44+/CD24− CSCs after irradiation and steroid hormone treatment. The percentages of CD44+/CD24− MCF10A cells were evaluated by flow cytometry three days after irradiation, after labeling with conjugated anti-human CD133−PE (phycoerythrin; Miltenyi Biotec) and CD44−FITC (Miltenyi Biotec). Hormonal treatment was performed two days before irradiation and every day afterwards. Pg: progesterone. Results are representative of three independent experiments. Error bars represent standard deviation. Asterisks denote significant differences (t-test, *p<0.05). (TIFF) [file pone.0077124.s001.tiff]
