# Supplementary material for: Generation of Breast Cancer Stem Cells by Steroid Hormones in Irradiated Human Mammary Cell Lines
Source: PLoS One. 2013 Oct 16;8(10):e77124. doi: 10.1371/journal.pone.0077124 (PMC3797732; doi:10.1371/journal.pone.0077124)
Supplement: Table S1 — microRNA expression levels in MCF10A cells, compared to control. For each experimental group (cells exposed to progesterone treatment alone, ALDH− cells and ALDH+ cells after irradiation and progesterone treatment), fold-changes (FCs) of miRNA expression were measured as compared to non-irradiated and non-treated control cells. If the expression ratios were >1, then FCs were equal to expression ratios. If the expression ratios were <1, then FCs were equal to the opposite of expression ratios. (PDF) [file pone.0077124.s002.pdf]

Table S1: microRNA expression levels in MCF10A cells, compared to control.

	**Fold change (comparing to control group)**		
microRNA	Progesterone	ALDH-	ALDH+
hsa-let-7a-5p	3.4019	5.8629	-2.0863
hsa-let-7b-5p	30.8164	17.8132	19.5919
hsa-let-7c	11.7259	4.7034	-1.6695
hsa-let-7d-5p	105.5679	7.6004	2.7566
hsa-let-7e-5p	9.7779	19.7112	1.0075
hsa-let-7f-5p	117.8952	19.3408	-1.3771
hsa-let-7g-5p	6.158	7.44	-1.2413
hsa-let-7i-5p	4.82	2.014	-1.3903
hsa-miR-1	112.5686	7.5538	406.4561
hsa-miR-100-5p	-2.4272	1.363	-4.5844
hsa-miR-107	13.5001	-1.439	-2.2449
hsa-miR-10a-5p	100.9428	11.6246	8.5204
hsa-miR-10b-5p	70.3752	4.7225	8.9983
hsa-miR-125b-5p	1.6052	1.9399	1.4118
hsa-miR-125b-1-3p	2.3963	-3.7597	1.1187
hsa-miR-128	1.2382	24.9663	-5.7327
hsa-miR-129-5p	108.807	7.8242	245.5867
hsa-miR-130a-3p	-11.0411	-10.9363	-10.1301
hsa-miR-130b-3p	-3.2984	-22.6707	-36.0254
hsa-miR-132-3p	10.7696	-1.2457	-3.0825
hsa-miR-140-5p	1.2999	-1.4177	-8.7927
hsa-miR-141-3p	1.8845	-2.2041	-3.1479
hsa-miR-145-5p	108.6262	-3.2956	-2.5195
hsa-miR-148a-3p	16.2315	3.5518	-1.2139
hsa-miR-152	3.4541	-1.0382	-2.629
hsa-miR-155-5p	112.5686	7.5538	14.3933
hsa-miR-15a-5p	-7.5098	-8.8109	-19.8479
hsa-miR-15b-5p	6.3731	-1.1599	-4.158
hsa-miR-16-5p	1.3493	-1.4006	-3.0506
hsa-miR-17-5p	-5.6383	-2.7471	-6.3378
hsa-miR-181a-5p	-5.6562	-17.197	-15.5936
hsa-miR-181b-5p	3.0315	-2.2839	-4.2874
hsa-miR-181c-5p	-2.2519	-3.9769	-2.8943
hsa-miR-181d	20.7585	1.5695	-3.2093
hsa-miR-182-5p	12.9241	1.1038	-3.3374
hsa-miR-186-5p	1.1997	1.0053	-1.6756
hsa-miR-18a-5p	-1.8231	-1.6468	-11.0769
hsa-miR-193b-3p	-1.7648	-4.4869	-1.7504
hsa-miR-195-5p	-12.7702	-4.2823	-28.3736
hsa-miR-199b-3p	51.9095	3.7679	6.6373
hsa-miR-199a-5p	554.2121	4.7498	9.0504
hsa-miR-19a-3p	-5.401	-4.6552	-7.9569
hsa-miR-19b-3p	-3.3688	-2.8836	-7.2644

hsa-miR-200a-3p	86.2585	5.7883	11.0292
hsa-miR-200b-3p	16.2375	1.1346	-2.8059
hsa-miR-200c-3p	16.4152	-1.1513	-5.3788
hsa-miR-202-3p	136.0973	7.5538	14.3933
hsa-miR-203a	40.8131	3.6425	5.2185
hsa-miR-204-5p	7187.102	4.3255	8.242
hsa-miR-205-5p	-6.811	-1.0212	-1.0733
hsa-miR-206	131.4583	7.5538	14.3933
hsa-miR-20a-5p	-1.0918	-2.3338	-4.0528
hsa-miR-20b-5p	1.4472	-1.547	-4.0359
hsa-miR-21-5p	28.3549	-1.371	-2.9061
hsa-miR-210	-1.979	-9.3094	-3.81
hsa-miR-212-3p	16.2315	1.0892	2.0754
hsa-miR-214-3p	49.8709	3.4984	6.3766
hsa-miR-22-3p	-11.0385	-2.2381	-4.3455
hsa-miR-222-3p	9.7227	1.0162	-2.2008
hsa-miR-223-3p	112.5686	7.5538	14.3933
hsa-miR-25-3p	18.1726	3.5505	1.0319
hsa-miR-26a-5p	4.8475	1.1407	-1.2695
hsa-miR-26b-5p	3.5722	-2.3334	-6.6063
hsa-miR-27a-3p	-6.7552	-1.9233	-3.2383
hsa-miR-27b-3p	1.949	-2.6862	-3.1406
hsa-miR-29a-3p	-2.123	-1.6303	-3.8651
hsa-miR-29b-3p	-11.9106	-10.0293	-4.9028
hsa-miR-29c-3p	-3.532	-1.5563	-3.3908
hsa-miR-31-5p	-4.9346	-2.3549	-2.6894
hsa-miR-328	33.8902	10.2713	1.98
hsa-miR-340-5p	3.6744	1.351	-2.2467
hsa-miR-424-5p	-1.2508	-1.5827	-9.6619
hsa-miR-429	112.5686	7.5538	14.3933
hsa-miR-485-5p	112.5686	7.5538	14.3933
hsa-miR-489	25.627	6.1813	7.1658
hsa-miR-495-3p	73.8092	4.9529	9.4374
hsa-miR-497-5p	3.7883	-2.341	29.7982
hsa-miR-548c-3p	126.3891	7.5538	14.3933
hsa-miR-607	112.5686	7.5538	14.3933
hsa-miR-613	112.5686	7.5538	14.3933
hsa-miR-7-5p	38.8783	-1.2069	-1.5448
hsa-miR-93-5p	141.2259	2.2276	-1.3169
hsa-miR-96-5p	2.9177	-1.2106	-1.1939
hsa-miR-98-5p	15.3872	2.2605	1.0491
cel-miR-39-3p	112.5686	7.5538	14.3933
